# Gel Rheological Properties and Storage Texture Kinetics of Starches Isolated from Anchote (*Coccinia abyssinica* (*Lam.*) *Cogn.*) Cultivars

**DOI:** 10.3390/gels9080631

**Published:** 2023-08-06

**Authors:** Yohannes Tolesa Wolde, Shimelis Admassu Emire, Workineh Abebe Zeleke, Felicidad Ronda

**Affiliations:** 1 School of Chemical and Bio-Engineering, Addis Ababa Institute of Technology, Addis Ababa University, King George VI Street, Addis Ababa P.O. Box 385, Ethiopia; 2 Department of Agriculture and Forestry Engineering, Food Technology, College of Agricultural and Forestry Engineering, University of Valladolid, 47002 Valladolid, Spain; 3 Ethiopian Institute of Agricultural Research, Addis Ababa P.O. Box 2003, Ethiopia

**Keywords:** anchote tuber, starch, hydration properties, gel, texture storage kinetics, rheological properties

## Abstract

Anchote is a tuber crop indigenous to Ethiopia. Starch hydration properties and important gel characteristics which include: color, gel rheological properties (at 2, 4, 6, 8, and 10% starch:water *w*/*w*) and gel texture evolution (at 10% starch:water *w*/*w*), during 0 to 192 h storage (at 4 °C), of anchote starches isolated from four anchote cultivars (Desta 01, Desta 24, white and red) were evaluated and compared with potato and cassava starches (PS and CS). The lightness (L*) and whiteness scores of the anchote starch ranged up to >95, with slight differences among the cultivars, making them pure starches. Swelling power (SP) and water solubility index (WSI) of the anchote starches increased with increasing cooking temperature (40, 50, 60, 70, 80 and 90 °C), and their rate of increase varied significantly with the control starches, as follows: CS < anchote starches < PS. Anchote starch gels resisted higher stresses before breaking their structure and showed higher elasticity with lower (tan δ)_1_ values than PS and CS gels. They also had greater viscoelastic moduli even at lower concentrations than the PS and CS gels, and their stability increased with increasing concentration. The study of the gels’ texture evolution during storage revealed that anchote starch gels had significantly higher (≥40%) initial and final (after 192 h) hardness and were less adhesive than the PS gel. Despite some significant differences in the studied starch gel quality parameters among the starches from the anchote cultivars, the results suggested their promising potential as additional new materials in the development of food products, specifically as a functional ingredient for the formulation of gel-like products.

## 1. Introduction

Starch is the most abundant polysaccharide in nature, and it is biodegradable, inexpensive, renewable, and widely available in numerous plants [1]. Starch exists in nearly all types of green plant tissues, including their leaves, fruits, pollen grains, roots, and stems. Starch is an essential dietary component and an important industrial product with numerous applications. The growing annual global consumption has increased the demand for new starch sources. Because of their high starch content, underutilized root and tuber crops have recently sparked considerable interest in starch extraction [1,2,3,4].

Anchote (*Coccinia abyssinica* (*Lam.*) *Cogn*.) is a native tuber crop cultivated in Ethiopia. It is an annual trailing vine belonging to the order *Cucurbitales* and the family *Cucurbitaceae.* Though anchote production and consumption have long been prevalent in western Ethiopia, its production and consumption have spread to the southern and southwestern regions of the nation [5,6,7]. The native users cultivate anchote for its medical, economic, nutritional, and socio-cultural benefits [1,8]. The anchote tuber has a significant number of beneficial nutrients, such as carbohydrates, protein, fat, fiber, and different minerals, with a total solids content that ranges between 25 and 28 g/100 g [9,10]. Compared to other root and tuber crops, this crop is renowned for its important protein and calcium content [6,9]. Anchote tubers have a high starch content (75–79%), making them a suitable alternative source of starch for use in several industries [1,5,7].

The food industry uses starches in a variety of ways to meet their technological requirements, including stabilizing, thickening, stiffening, gelling, water-holding, binding, and bulking agents to maintain and enhance the textural attributes of food products [1,11]. Food with added starch has better physical and functional characteristics, as well as a better texture and mouthfeel [12]. However, the physicochemical qualities of starch, such as its structure, particle size, crystallinity, amylose content, and intrinsic characteristics, like hydration and thermal properties, could determine its application [4,13]. Amylose and amylopectin levels in starch also govern starch functionality in many applications [11]. Amylose retrogrades easily and forms tough gels, while amylopectin is more stable and creates soft gels [14]. During heat processing, the ratio of amylose in starch and the branching characteristics of the amylopectin molecules can have an impact on the starch pasting and gel texture characteristics [15].

Hence, in-depth characterization of the starches available in these underexploited root and tuber crops is crucial for their use in food processing. The physicochemical characteristics of starches obtained from different cultivars of roots and tubers were studied, and it was reported that they had different amylose, and phosphorus contents, particle size distribution, granule sizes, enzyme digestibility, and functional, thermal and pasting properties. For example, the starches of 11 different sweet potato cultivars varied in their mean granule sizes and distributions; pastes of high amylose content starches also showed a higher tendency to retrograde [4]. According to Chisenga et al. [16], variations in physicochemical properties among cassava varieties were caused by differences in amylose, lipid, protein content, and starch granule size distribution. The white, yellow, and purple sweet potato starches had different granule sizes and amylose contents, but all had the same crystalline structure; furthermore, starches from the same or different colored varieties also had differences in their swelling power, water solubility, gelatinization temperature, pasting viscosity, and digestion properties [17]. According to the findings of Ren et al. [18], ramie root starch differed significantly from rice starch in terms of starch component, crystalline structure, and functional qualities while being just marginally more comparable to potato starch. As a result, ramie starch showed promise as a potato starch substitute. The rheological and texture properties of various starch gels obtained from different crops were also reported. For example, according to Zhang et al. [15], amylose has a significant impact on the pasting and gel texture characteristics of chestnut starch during cooking. On the other hand, arrowroot starch gel texture showed a sharp rise in the gel hardness of all starches after 72 h of storage [19].

In the prior pasting characteristics study, anchote starches demonstrated greater resistance to heat and shear force as well as a higher final viscosity than PS and CS. Gels from anchote starch also showed rheological properties of true gels, with notably lower (tan δ)_1_ values and considerably larger viscoelastic moduli than PS and CS gels [3]. However, there is still a dearth of studies on important starch gel intrinsic characteristics like storage texture kinetics and the effect of starch concentration on gel rheological properties of anchote starch gel. Hence, this study was undertaken to evaluate the starch hydration properties and color, rheological, and textural characteristics of anchote starches that were isolated from four cultivars and compare them with the common tuber starches (potato and cassava starches).

## 2. Results and Discussion

### 2.1. Strach Swelling Power (SP) and Water Solubility Index (WSI)

**Figure 1:**
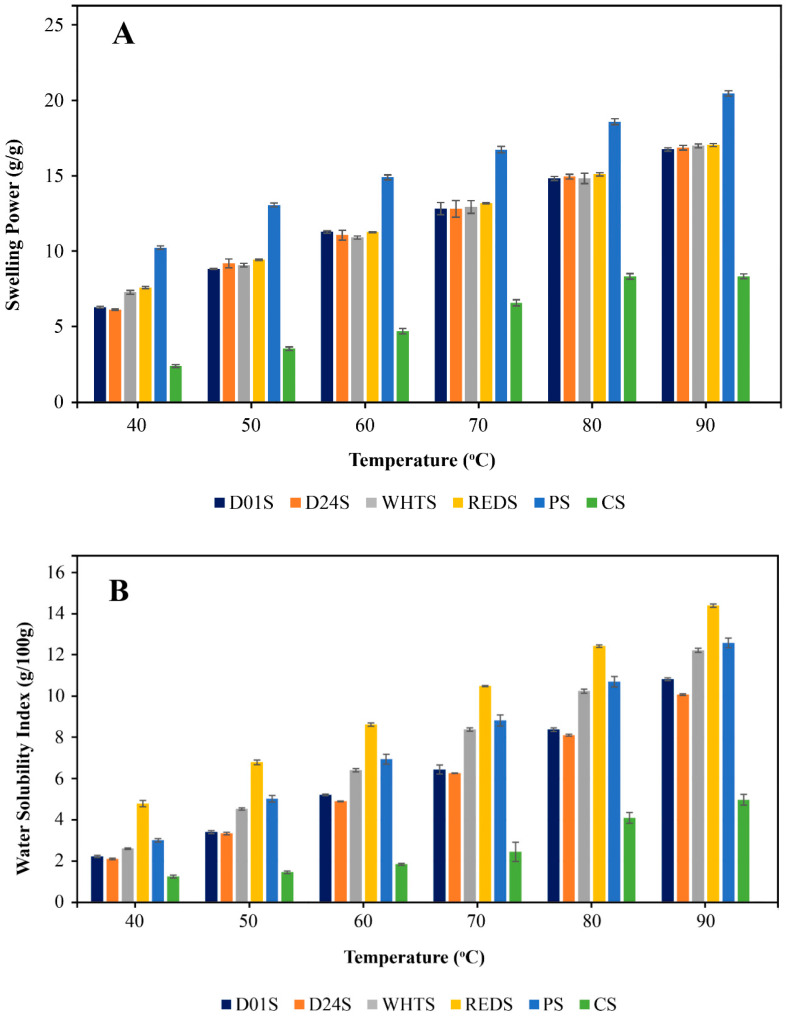
Swelling power (**A**) and water solubility index (**B**) of the starches. D01S, D24S, WHTS, and REDS, respectively, represent Desta 01, Desta 24, white, and red anchote cultivar starches. PS = potato starch and CS = cassava starch. The error bars indicate the standard deviation.

The results of starch hydration properties determination, swelling power (SP) and water solubility index (WSI), were performed by heating the starch–water suspensions at temperatures ranging from 40 to 90 °C at 10 °C intervals, are presented in Figure 1. Starches from the anchote cultivars showed significant differences (*p* < 0.05) in their SP and WSI among themselves and with PS and CS. Increasing cooking temperature significantly raised the SP and WSI of all the starches studied. The SP of anchote cultivars ranged from 6.12 to 17.04 g/g for D24S (at 40 °C) and REDS (at 90 °C), respectively. Among the anchote starches, the SP of REDS was the highest at all temperature levels, and this could indicate its relatively higher water absorption capacity than the starches from the remaining anchote cultivars. In addition, at all temperatures, the SPs of anchote starches were significantly higher and lower than the SPs of PS and CS, respectively. WSI reveals the degree of dissolution during the starch swelling process. The WSI of anchote cultivars varied from 2.10 g/100 g (D24S at 40 °C) to 14.41 g/100 g (REDS at 90 °C) standing between the WSIs of CS and PS, as follows: CS < anchote starch ≤ PS. At all of the cooking temperature levels, the WSI of REDS was the highest, followed by WHTS and D01S, while those of D24S were the lowest. It has been reported that the higher phosphate group contents led to higher SP and solubility of starches obtained from potato cultivars [20]; therefore, it is possible that the higher SP and WSI of anchote starches are caused by their higher phosphorus content. According to a previous report [3], WHTS and REDS had higher phosphorus contents (93.3 and 92.2 mg/100 g, respectively), and could be the likely reason they had higher SP and WSI. Additionally, SP and WSI are affected by variations in granule morphology, particle size, amylose content, and the crystallinity of starches [3,18,21]. The WSI of PS ranged from 3.01 g/100 g to 12.60 g/100 g, which was comparable to the result of anchote starches; however, the WSI of CS starch varied from 1.24 g/100 g to 4.98 g/100 g, and it was significantly lower than PS and anchote starches. Tessema and Admassu [7] reported that the water solubility of potato and anchote starches at 95 °C was 22.5% and 19.5%, respectively, which was higher than the findings in this study. This could be due to several factors, such as the differences in the cultivar type, starch isolation methods, and cooking temperatures employed [16,17].

### 2.2. Color Measurements

Color is a vital criterion in assessing starch quality [4]. The result revealed significant differences (*p* < 0.05) in the color attributes of starches from the anchote cultivars (Table 1). The lightness (L*) of the starches of the anchote cultivars varied from 95.40 to 97.72. The L* score of REDS gel was significantly (*p* < 0.05) lower than the gels from the remaining anchote starches and the reference starches. In addition, the gels from reference starches had significantly higher L* values than the anchote starch gels. However, all the starches of anchote cultivars had L* values greater than 90, validating their purity and acceptance as pure starches [7]. The a* values of the studied starches varied between 0.016 (D24S) and 0.207 (REDS). The a* value scored by REDS was significantly (*p* < 0.05) higher than the rest of the anchote cultivars, PS and CS, which had almost equivalent scores. This underlines the relatively more reddish-color in REDS due to the more reddish-colored tuber. Similarly, the b* values of the studied anchote cultivars varied significantly between 0.827 (D01S) and 1.433 (REDS). In contrast, the b* value of PS (0.630) and CS (0.793) was lower than the starches of anchote cultivars. This implies that anchote starches had higher yellowness compared to PS and CS starches. The yellowness (b*) of the studied starches varied in the following order: PS < CS < D01S < WHTS < D24S < REDS. The whiteness (W) of anchote starches varied considerably from 95.17 to 97.57. The PS and CS gels had significantly higher W than the anchote gels, while REDS gel scored significantly (*p* < 0.05) lower W than the rest of the anchote starch gels. The result obtained suggests that anchote starches had acceptable color characteristics comparable with PS and CS. The values of the anchote starch color attributes recorded were relatively higher than those described by Tessema and Admassu [7], where L*, a*, and b* were 89.8, 0.025, and 3.63, respectively. This variation could be because of the type of cultivars used, and the starch extraction method followed [7].

### 2.3. Gel Rheological Properties

The gel rheological properties (strain sweeps and frequency sweeps) were studied, and the findings are shown in Table 2. Gels made from anchote cultivar starches exhibited a comparable physical appearance to PS and CS starch gels; however, anchote starches had thicker gels in all concentrations (from 2 to 10 g starch/100 g dispersion).

#### 2.3.1. Strain Sweeps

The results of dynamic oscillatory tests carried out at 25 °C on gels obtained from starch-water dispersions at different concentrations (from 2 to 10 g starch/100 g dispersion) are presented in Table 2. The strain sweeps were performed to determine the stress at the cross point, where G′ = G″ and tan δ = 1, which denotes the stress where the rheological behavior of the gels changes from a predominantly elastic to a predominantly viscous character [22]. It also allowed us to obtain the maximum stress that the gels can tolerate without breaking (τ_max_) and create the linear viscoelastic region (LVR). The results revealed that the effects of starches of botanical origin and concentration were significant (*p* < 0.05) on both τ_max_ and stress at the cross point and, as expected, increased with starch concentration levels. The cross point and τ_max_ of PS and CS starch gels prepared at concentrations above 6% were above the maximum values applied during the test, denoting their high strength and stability within the studied strain range (0.10 to 1000%); this finding was in accordance with previous reports [3,23]. However, at concentrations of <6%, anchote starches had significantly higher stress at cross-over point and τ_max_ scores than PS and CS starch gels. Thus, anchote starches could be considered as a good option to obtain gels with stable structures at lower concentrations.

#### 2.3.2. Frequency Sweeps

The fitted values of frequency sweep to the power law model demonstrated the significant (*p* < 0.05) individual and combined effects of starch botanical sources and concentration levels on the viscoelastic moduli scores of the gels (Table 2). This could be due to the strong dependence of starch gel rheological properties on branch chain length distribution of amylopectin, level of amylose, and starch chemical composition [3,21,24].

The G_1_′ and G_1_″ increased with increasing starch concentration levels within the studied frequency range, and the values of G_1_′ were higher than that of G_1_″, with tan δ < 1, which showed the solid-like behavior of the gels (Table 2). However, the degree of increase varied differently among the anchote cultivars themselves and with the PS and CS gels. The effect of concentration on the elastic modulus of D24S gels was more marked than in the rest of anchote cultivar starches, which exhibited similar patterns of moduli increase. At all concentrations, gels from the anchote cultivars starches had significantly (*p* < 0.05) higher elastic and viscous moduli and noticeably low (tan δ)_1_ values compared to PS and CS gels. The reliance of the elastic and viscous moduli on the frequency of oscillation (*a* and *b* exponents) was also significantly lower in anchote starch gels than in PS and CS gels, indicating anchote starch had more consistent and structurally stable gels. The dominance of anchote starch gel elastic behavior on the viscous behavior regardless of cultivar, even at low concentration levels, which shows their higher strength than PS and CS gels [23], underlining the potential of anchote starch as a good functional ingredient for food product development, mainly in the formulation of gel-like products.

### 2.4. Gel Storage Texture Kinetics

**Figure 2:**
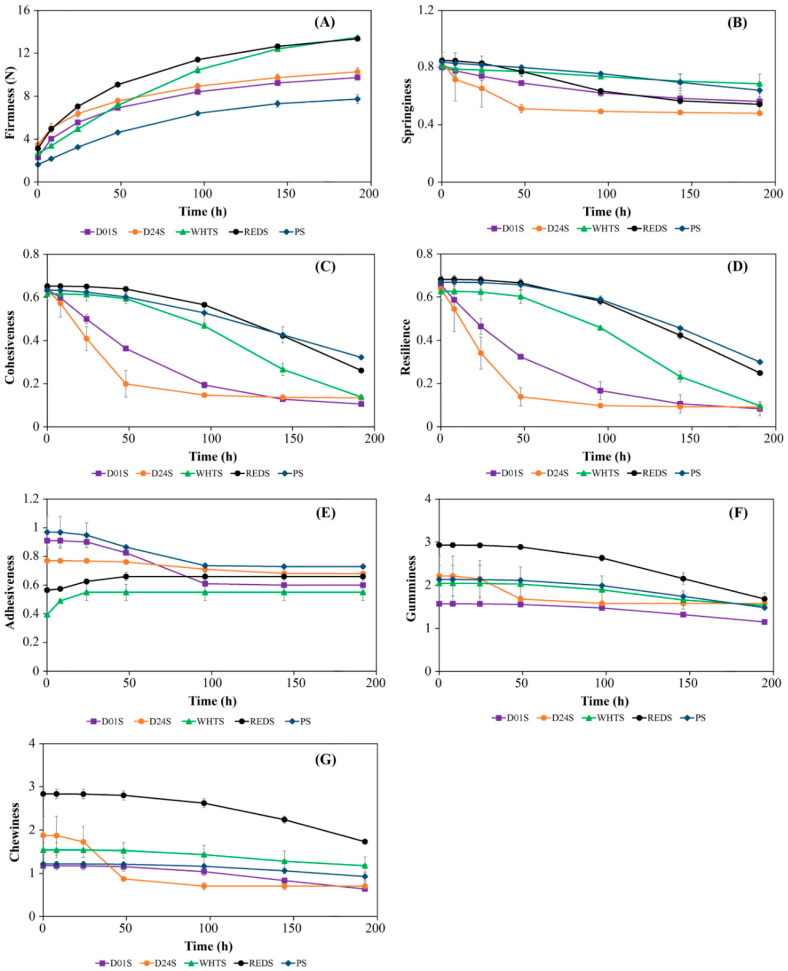
Texture evolution for the starch gels stored at 4 °C: Firmness (**A**), springiness (**B**), cohesiveness (**C**), resilience (**D**), adhesiveness (**E**), gumminess (**F**) and chewiness (**G**). Anchote starches D01S, D24S, WHTS, and REDS, respectively, represent Desta 01, Desta 24, white, and red anchote cultivar starches. PS = potato starch.

The evolution of gel texture properties with storage time (from 0 to 192 h) at 4 °C of gels made from the four anchote starches and PS is presented in Figure 2. The kinetic parameters achieved from fitting this evolution to the Avrami model are shown in Table 3. Only potato starch gel was used as a reference because cassava starch did not form a self-standing gel at 10% concentration.

Firmness is the quantity of force needed to make a given amount of deformation [11]. The initial firmness of the studied anchote starches varied between 2.302 N (D01S) and 3.472 N (D24S). The anchote starches had 40–110% higher initial firmness than the PS gel. A similar trend was observed in the G_1_′ of the gels obtained in gel viscoelastic property determination discussed earlier. The increase in terms of firmness with storage time was observed in all of the studied gels (Figure 2A), and this could be mainly related to amylopectin recrystallization [25,26]. Variations were also observed in the rate of firmness increase with storage time among the starches of anchote cultivars themselves and with gels from PS, which could be attributed to the high dependence of amylopectin recrystallization process, which is greatly reliant on the starch composition and botanical origin [27]. The hardening kinetics of the gels can be directly evaluated from *k* or *t*_1/2_. The result of half-life (*t*_1/2_) showed that the firmness gels from D01S leveled off much faster than the other starch gels because D01S gel needed only 41.89 h to pass from *P*_0_ to *P_∞_*_/2_, while the WHTS gel took 67.38 h (which was the highest). This could indicate that the products made from D01S gels will form stable gels faster than the rest of anchote cultivars [28]. The total increase in hardness during storage (*P_∞_* − *P*_0_) was significantly (*p* < 0.05) higher in anchote starches compared to potato starch (PS).

Springiness is a mechanical textural characteristic that shows the quickness and degree of retrieval from a deforming force [27]. The initial springiness of anchote starch gels varied between 0.801 and 0.850 for D01S and REDS, respectively, and showed no appreciable differences. The springiness of the studied starch gels decreased significantly (*p* < 0.05) with storage time, but the rate of decrease among the anchote cultivars varied noticeably. The springiness of D24S dropped significantly up to 48 h storage time, but later the value remained almost constant (Figure 2B). This could be due to the higher amylose content of D24S compared to other anchote cultivars [3]. In contrast, the springiness of WHTS decreased at a very slower rate, as shown in Figure 2B. The studied starches of anchote cultivars had similar springiness characteristics with PS. The rate constant (*k*) of D24S (0.017 h^−n^) was greater than the other studied starches (Table 3). The half-life time (*t*_1/2_) of the studied anchote starches varied between 21.74 and 93.81 h. The D24S and WHTS anchote cultivars had the lowest *t*_1/2_, indicating that their springiness stabilized much faster than the other anchote cultivars. The springiness of the gel from PS took a significantly longer time than the anchote starch gels because it had significantly higher *t*_1/2_ (100.97 h) compared to the anchote starch gels.

The initial cohesiveness of the cultivars of anchote starch gels varied from 0.616 to 0.652 without significant (*p* < 0.05) differences among them. However, over the studied storage time, the cohesiveness of the gels decreased significantly, as shown in Figure 2C. Avrami model parameters showed that the *k* values were not significantly different in all studied starch gels. D24S had the lowest *t*_1/2_ (24.71 h) value, which was significantly (*p* < 0.05) different from the other anchote cultivars. The results also revealed that the loss of cohesiveness with storage time was faster in D24S gels than in the rest of the anchote cultivars and PS.

Resilience is a textural parameter that expresses the instant springiness and how well the product returns to its previous position [28]. The initial resilience scores of anchote starch gels varied between 0.628 (WHTS) and 0.683 (REDS) and were not significantly different (*p* < 0.05). However, the values significantly (*p* < 0.05) decreased with storage time, and the starch gels from the four anchote cultivars had different characteristics in resilience over storage time. The resilience of D01S and D24S decreased very much between 0 and 48 h storage periods, and later a gradual decrease was observed (above 48 h), as shown in Figure 2D. In contrast, the resilience of WHTS and REDS was almost constant between 0 and 48 h storage periods and significantly decreased above a 48 h storage period (Figure 2D). The study revealed that WHTS and REDS had very high resilience up to 2 days (48 h) storage period compared to the other cultivars. The Avrami relation was fitted, and the recorded *k* values of all anchote gels were not significantly (*p* < 0.05) different. In contrast, the resilience half-life times (*t*_1/2_) of the studied gels varied significantly. The fastest decrease in resilience was obtained in D24S and D01S gels, having *t*_1/2_ values of 18.83 h and 40.52 h, respectively. Thus, this confirms the presence of faster kinetics in these gels. On the other hand, REDS had the highest *t*_1/2_ (98.89 h) among the studied cultivars, which underlined the slower decrease in the resilience of this gel over storage time. The PS gels had comparable results with REDS, as shown in Figure 2D.

Adhesiveness is more of a surface characteristic and depends on a combined effect of adhesive and cohesive forces, and others include viscosity and viscoelastic as well [29]. The initial adhesiveness of anchote starch gels varied from 0.395 to 0.910 for WHTS and D01S, respectively. The adhesiveness of the gels from WHTS and REDS increased over the storage time, as shown in Figure 2E, and between 0 and 24 h, the adhesiveness of WHTS gels increased significantly (*p* < 0.05), and later, the rate of increase was very small for the rest of storage periods. A similar trend was observed for REDS gels, except at the beginning, where the adhesiveness increased between 0 and 48 h storage periods. On the other hand, starch gels made from D01S significantly decreased throughout the storage periods. Similar characteristics were observed in PS gels, as shown in Figure 2E. The fitted value of the Avrami equation shows that D24S gels had the highest *k* value (0.018 h^−n^), while WHTS had the lowest *t*_1/2_ (7.64 h) value. The *t*_1/2_ values of studied starch gels varied considerably in the following order: WHTS (7.64 h) < D24S (20.59 h) < REDS (22.01 h) < PS (41.23 h) < D01S (68.41 h).

Gumminess, which is frequently used to describe the amount of energy needed to break down semi-solid foods, is closely tied to a trained panel’s sensory assessment [30]. The initial gumminess of anchote starch gels varied between 1.566 (D01S) and 2.935 N (REDS). The REDS gel had significantly (*p* < 0.05) higher initial gumminess, while the other anchote cultivars (D01S, D24S and WHTS) were statistically not significant (*p* < 0.05). The gumminess of anchote starch gels decreased significantly (*p* < 0.05) over the studied storage time, as shown in Figure 2F. Similar characteristics were observed in PS gel, in which PS had initial gumminess (2.134 N) comparable to the anchote cultivars, and it significantly decreased over the stored period. The Avrami relation was also well-fitted. The D24S had the highest *k* value and the lowest *t*_1/2_ value implying D24S gels had the fastest decrease in gumminess over the storage time. Generally, the studied starch gels *t*_1/2_ values varied in the following order: D24S (23.20 h) < WHTS (98.26 h) < REDS (108.76 h) < D01S (113.85 h) < PS (149.61 h).

The quantity of energy needed for masticating a semi-solid sample to a steady state of swallowing is represented by chewiness [29]. The initial chewiness of anchote starch gels varied significantly from 1.172 (D01S) to 2.834 N (REDS). The initial chewiness of PS (1.216 N) gel showed no significant difference with D01S gel but was significantly different (*p* < 0.05) compared to the three anchote cultivars. Generally, the chewiness of the studied starch gels showed a similar trend with their gumminess, in which the values decreased during the studied storage period (Figure 2G).

In this study, slight variations were observed in the textural properties of the studied anchote starch gels, and they were more or less comparable with PS gels. The differences in the textural properties among the starch gels from the anchote cultivars could be due to their variable chemical compositions and amylose contents [15]. According to Marta et al. [31], non-starch components also decrease the texture characteristics of starches. Thus, the various texture characteristics seen in anchote cultivars’ starch gels could be attributed to differences in their non-starch components.

## 3. Conclusions

Starches isolated from four cultivars of anchote tubers were examined and compared to potato and cassava starches. The result revealed the presence of significant variations in the hydration, rheological, and textural properties of the starch gels. According to the findings, anchote starch had acceptable color characteristics that were similar to those of potato and cassava starches. The lightness (L*) and whiteness scores of the anchote starch ranged >95, with slight differences among the cultivars, meaning that they can be considered pure starches. When heated in water at various temperatures, the examined anchote cultivars displayed distinct SP and WSI, and a significant difference (*p* < 0.05) was observed in comparison to potato and cassava starches. Anchote starch gels resisted higher stresses before breaking their structure and showed higher elasticity with lower tan δ values than PS and CS gels. They also showed higher viscoelastic moduli even at lower concentrations than the PS and CS gels, and their stability increased with increasing concentration. Gel texture storage (at 4 °C) kinetics study revealed that anchote starch gel had significantly higher (≥40%) initial firmness and final hardness after 192 h of storage and relatively less adhesive than PS. The difference in the texture characteristics observed in anchote starch gels could be attributed to differences in their non-starch components. Despite some significant differences in the studied starch gel quality parameters among the starches from the anchote cultivars, the results suggested their promising potential as additional new materials in the development of food products, specifically as a functional ingredient for the formulation of gel-like products.

## 4. Materials and Methods

### 4.1. Materials

In this study, four anchote cultivars were used. Desta 01 (D01) and Desta 24 (D24) anchote tubers were acquired from Debre Zeit Agricultural Research Center (DZARC), Ethiopia, while white (WHT) and red (RED) are local anchote cultivars grown locally in the western part of Ethiopia. Potato (PS) and cassava (CS) starches were references supplied by Ferrer Alimentación S.A. (Barcelona, Spain) and Cargill S.L. (Brenntag, Seville, Spain), respectively.

### 4.2. Isolation of Starch

Anchote starch was isolated following the previous method [3]. The anchote tubers were sorted, washed, and hand-peeled. The peeled anchote tubers were cut into small cubes and blended with distilled water at a ratio of 1:10 (*v*/*v*) for 4 min using a laboratory-grade blender (Silver Crest multifunctional blender, Shandong, China). The slurry was separated by passing through a thin cotton cloth. The filtrate was held at room temperature for 14 h before the supernatant was drained, and the residue was washed repeatedly with distilled water until the color became white. The sediment was dried in a drying oven at 45 °C for 24 h. The dried native starch was milled, packed in a zip-locked polythene bag, and kept for further analysis. The isolated anchote starches were denoted as Desta 01 starch = D01S, Desta 24 starch = D24S, White starch = WHTS, and Red starch = REDS.

### 4.3. Starch Swelling Power (SP) and Water Solubility Index (WSI)

The water solubility index (WSI) and swelling power (SP) of starch samples at different temperatures were measured following the method of Abegunde, Mu, Chen, and Deng [4] with slight modifications. In brief, 0.28 g (W_0_) of the starch samples were dispersed in 10 mL of distilled water in pre-weighed centrifuge tubes. The starch–water suspension was heated in a water bath at different temperatures (40, 50, 60, 70, 80, and 90 °C for 30 min), subsequently cooled to room temperature and centrifuged at 3000× *g* for 10 min. The supernatant was dried overnight at 110 °C to obtain its solid content (W_1_), and the weight of the sediment was recorded (W_2_). The hydration properties WSI and SP were calculated using the following equations:

(1) WSIg100g=W1W0×100

(2) SPgg=W1W0−W1

### 4.4. Color Measurements

The starch color was measured using a Hunter Lab Colorimeter (3nh Technology Co., Ltd., Shenzhen, China). The results were achieved in the CIE L* a* b* coordinates using the D65 standard illuminant and 10° standard observer. The L*, a* and b* color values indicates lightness from black (0) to white (100), green (−) to red (+), and blue (−) to yellow (+), respectively [32]. The whiteness (W) of starches was determined as per the method described by Kale et al. [33] using Equation (3).

Whiteness (W) = 100 − [(100 − L*)^2^ + a*^2^ + b*^2^]^1/2^ (3)

The starch samples were measured eight times.

### 4.5. Gel Rheological Properties

The dynamic oscillatory studies on the starch gels were carried out with a Kinexus Pro+ rheometer (Malvern Instruments Ltd., Malvern, UK) with a serrated parallel plate geometry of 40 mm diameter and a measurement gap of 1 mm. Starch gels were prepared using Rapid Visco Analyser (RVA 4500, Perten Instruments, PerkinElmer, Sydney, Australia) according to the method of [28] with some modifications. The suspensions of starch in water at different concentrations (2, 4, 6, 8 and 10 g starch/100 g dispersion with 27 g total weight) were made. Briefly, the starch–water mixture was stirred with a constant rotating paddle at 160 rpm, heated from 50 to 95 °C at a rate of 6 °C/min and held at 95 °C for 15 min. To allow the sample relaxation, the starch gels were placed on the bottom of the plate and left there for five minutes. Strain sweeps were performed at a constant frequency of 1 Hz from 0.10 to 1000% strain. Frequency sweeps from 10 to 1 Hz were performed at a constant strain of 0.5%, which was in the linear viscoelastic region (LVR). The tests were carried out at a fixed temperature of 25 °C. The rheological data were examined using rSpace rheometry software for Kinexus V1.72 (Malvern Instruments Ltd., UK). Frequency sweep data were fitted to the power law model as described by Ronda, Villanueva, and Collar [34]. At a frequency of 1 Hz, the recorded viscoelastic parameters, G_1_′ and G_1_″, and (tan δ)_1_, indicate the elastic and viscous moduli, as well as the loss tangent. The exponents of the associated potential equations (Power-law model) are a, b, and c, and they quantify the dependency of the dynamic moduli on the oscillation frequency. All gels and tests were performed in triplicate.

### 4.6. Gel Storage Texture Kinetics

Starch gel texture properties were analyzed, as described in Abebe and Ronda [28], with slight modification. The gels were prepared in a Rapid Visco Analyser (RVA) canister from 10% (*w*/*w*) suspensions of starches (27 g total weight) using RVA (RVA 4500, Perten Instruments, PerkinElmer, Sydney, Australia), according to the method used in Section 4.5 above. Then, the gels were cooled to room temperature, hermetically sealed, and stored for 0, 8, 24, 48, 96, 144, and 192 h at 4 °C. The samples were equilibrated to room temperature for 1 h before texture analysis. The texture properties of firmness, adhesiveness, springiness, cohesiveness, gumminess, chewiness, and resilience of starch gels were evaluated using a TA-XT2 Texture Analyser (Stable Microsystems, Surrey, UK) provided by the software Texture Expert. Measurements were performed on gels of 2 cm diameter and 2 cm height. A Texture Profile Analysis (TPA) double compression test was performed using a 75 mm diameter aluminum probe (SMSP/75), and 1 mm/s speed was used. A 50% deformation was preferred to avoid the total damage to the gel structure in the first compression of TPA tests [28]. All samples were analyzed at least in duplicates in two different gels.

The data of texture evolution with time was fitted with the Avrami equation, as utilized in Abebe and Ronda [28]:

(4) $$\frac{P_{\infty }-P_{t}}{P_{\infty }-P_{0}}=e^{-kt^{n}}$$

where *P* represents the texture properties (firmness, adhesiveness, springiness, cohesiveness, gumminess, chewiness, or resilience) at initial time (*P*_0_), at infinite time (leveling-off value), (*P*_∞_) and the value at any time *t*, (*P*_t_); *k* is a constant of velocity and *n* is the Avrami exponent. The values of the constants *k* and *n* were used to calculate the value of half-life, *t*_1/2_, which is the time required to achieve 50% of leveling-off extent:

(5) t12=−ln⁡0.5k1n

### 4.7. Statistical Analysis

The results were reported as means and corresponding standard deviations. One-way analysis of variance (ANOVA) was carried out followed by Tukey’s honest significant difference (HSD) test to establish the least significant difference (LSD) at *p* < 0.05. Also, two-way ANOVA was performed using Statgraphics Centurion v.18 (Bitstream, Cambridge, MN, USA).

## Figures and Tables

**Table 1:** Color characteristics of anchote cultivars, potato, and cassava starches.

Samples	L*	a*	b*	Whiteness (W)
D01S	97.72 ± 0.31 cd	0.018 ± 0.007 a	0.827 ± 0.047 ab	97.57 ± 0.30 cd
D24S	96.78 ± 0.23 b	0.016 ± 0.002 a	1.007 ± 0.051 b	96.63 ± 0.23 b
WHTS	97.11 ± 0.14 bc	0.030 ± 0.010 a	0.917 ± 0.055 b	96.97 ± 0.13 bc
REDS	95.40 ± 0.49 a	0.207 ± 0.015 b	1.433 ± 0.215 c	95.17 ± 0.47 a
PS	98.09 ± 0.30 d	0.027 ± 0.006 a	0.630 ± 0.061 a	97.99 ± 0.28 d
CS	98.53 ± 0.19 d	0.033 ± 0.006 a	0.793 ± 0.015 ab	98.33 ± 0.17 d

D01S, D24S, WHTS, and REDS, respectively, represent Desta 01, Desta 24, white, and red anchote cultivar starches. PS = potato starch and CS = cassava starch. L*, a*, b* were the CIE LAB color coordinates; Whiteness (W) = 100 − [(100 − L*)^2^ + a*^2^ + b*^2^]^1/2^. Results are expressed as mean values ± standard deviations. Values in the same column followed by the same letter are not significantly different (*p* < 0.05).

**Table 2:** Effect of starch concentration (g starch/100 g dispersion) on the rheological properties of the starches from anchote cultivars and the reference starches.

Sample	Concentration	G_1_′ (Pa)	a	G_1_″ (Pa)	b	(tan δ)_1_	c	Cross Point (Pa)	τ_max_ (Pa)
D01S	2	25 ± 4 fg	0.13 ± 0.02 bcd	5.70 ± 0.92 cd	0.42 ± 0.01 i	0.23 ± 0.01 cdef	0.30 ± 0.02 h	21 ± 3 abc	0.1 ± 0.1 a
	4	71 ± 1 i	0.13 ± 0.01 bcd	15.41 ± 0.25 j	0.29 ± 0.01 a	0.22 ± 0.01 cde	0.15 ± 0.01 abcdef	108 ± 1 f	24 ± 1 a
	6	62 ± 7 h	0.16 ± 0.02 bcd	15.34 ± 1.49 ij	0.32 ± 0.01 bcde	0.25 ± 0.01 def	0.16 ± 0.01 abcdefg	225 ± 8 h	128 ± 14 c
	8	75 ± 2 ij	0.16 ± 0.01 bcd	20.03 ± 0.39 kl	0.34 ± 0.01 def	0.27 ± 0.01 f	0.18 ± 0.01 cdefg	467 ± 2 j	305 ± 10 de
	10	108 ± 11 mn	0.13 ± 0.01 bcd	26.44 ± 2.79 m	0.34 ± 0.01 ef	0.24 ± 0.01 def	0.21 ± 0.01 fg	772 ± 51 l	608 ± 50 g
D24S	2	23 ± 1 efg	0.10 ± 0.02 b	3.73 ± 0.18 b	0.52 ± 0.01 k	0.16 ± 0.01 a	0.43 ± 0.03 i	5 ± 1 a	1 ± 1 a
	4	115 ± 2 no	0.11 ± 0.01 b	18.78 ± 0.33 k	0.31 ± 0.01 b	0.16 ± 0.01 a	0.20 ± 0.01 efg	92 ± 1 ef	31 ± 1 a
	6	96 ± 17 l	0.14 ± 0.01 bcd	21.81 ± 3.73 l	0.31 ± 0.01 b	0.23 ± 0.01 cdef	0.16 ± 0.01 abcdefg	305 ± 11 i	149 ± 24 c
	8	103 ± 1 lm	0.15 ± 0.01 bcd	25.57 ± 0.19 m	0.32 ± 0.01 bcde	0.25 ± 0.01 def	0.17 ± 0.01 bcdefg	545 ± 1 k	375 ± 10 f
	10	130 ± 3 p	0.14 ± 0.01 bcd	31.13 ± 0.20 o	0.33 ± 0.01 cdef	0.24 ± 0.01 def	0.20 ± 0.01 efg	854 ± 11 n	784 ± 6 i
WHTS	2	25 ± 1 fg	0.14 ± 0.02 bcd	4.91 ± 0.23 bc	0.44 ± 0.01 ij	0.19 ± 0.01 abc	0.30 ± 0.03 h	9 ± 1 ab	2 ± 1 a
	4	77 ± 4 ij	0.12 ± 0.01 bc	13.58 ± 0.81 ghi	0.33 ± 0.01 cdef	0.17 ± 0.01 ab	0.21 ± 0.01 fg	70 ± 3 de	29 ± 1 a
	6	80 ± 3 jk	0.13 ± 0.01 bcd	16.66 ± 0.75 j	0.32 ± 0.01 bcd	0.21 ± 0.01 bcd	0.19 ± 0.01 defg	207 ± 6 gh	79 ± 3 b
	8	78 ± 1 ij	0.16 ± 0.01 bcd	19.54 ± 0.35 k	0.34 ± 0.02 f	0.25 ± 0.01 ef	0.18 ± 0.01 cdefg	458 ± 13 j	271 ± 9 d
	10	120 ± 9 o	0.14 ± 0.01 bcd	28.66 ± 1.92 n	0.34 ± 0.01 def	0.24 ± 0.01 def	0.20 ± 0.01 efg	819 ± 83 m	676 ± 84 h
REDS	2	23 ± 1 efg	0.13 ± 0.01 bcd	4.39 ± 0.22 bc	0.45 ± 0.01 j	0.19 ± 0.01 abc	0.32 ± 0.02 h	7 ± 1 a	2 ± 1 a
	4	73 ± 1 ij	0.11 ± 0.01 b	12.50 ± 0.13 g	0.33 ± 0.01 cdef	0.17 ± 0.01 ab	0.22 ± 0.01 g	58 ± 1 d	28 ± 1 a
	6	77 ± 3 ij	0.13 ± 0.01 bcd	14.89 ± 0.69 hij	0.33 ± 0.01 cdef	0.19 ± 0.01 abc	0.20 ± 0.01 efg	184 ± 3 g	81 ± 4 b
	8	87 ± 5 k	0.14 ± 0.01 bcd	20.57 ± 0.90 kl	0.34 ± 0.01 ef	0.24 ± 0.01 def	0.20 ± 0.01 efg	436 ± 10 j	321 ± 22 e
	10	129 ± 9 p	0.13 ± 0.01 bcd	29.14 ± 1.41 n	0.34 ± 0.01 f	0.23 ± 0.01 cdef	0.21 ± 0.01 fg	822 ± 52 mn	617 ± 109 g
PS	2	9 ± 1 abc	0.20 ± 0.02 de	3.36 ± 0.15 b	0.32 ± 0.02 bcd	0.38 ± 0.01 h	0.12 ± 0.03 abc	7 ± 1 a	1 ± 1 a
	4	11 ± 2 bc	0.27 ± 0.04 ef	5.15 ± 0.42 bc	0.37 ± 0.03 gh	0.46 ± 0.04 ij	0.11 ± 0.04 a	48 ± 5 cd	2 ± 1 a
	6	17 ± 1 cdef	0.24 ± 0.01 ef	7.95 ± 0.36 e	0.37 ± 0.01 gh	0.46 ± 0.02 ij	0.13 ± 0.01 abcd	ND	ND
	8	30 ± 1 g	0.25 ± 0.01 ef	13.33 ± 0.40 gh	0.36 ± 0.01 g	0.45 ± 0.01 i	0.11 ± 0.01 ab	ND	ND
	10	80 ± 9 jk	0.19 ± 0.01 cde	25.79 ± 2.50 m	0.32 ± 0.01 bc	0.32 ± 0.01 g	0.12 ± 0.01 abcd	ND	ND
CS	2	1 ± 1 a	−0.39 ± 0.24 a	0.73 ± 0.01 a	0.59 ± 0.05 l	0.59 ± 0.12 l	0.92 ± 0.19 j	0.07 ± 0.01 a	0.01 ± 0.01 a
	4	6 ± 1 ab	0.24 ± 0.04 ef	3.34 ± 0.12 b	0.38 ± 0.01 h	0.52 ± 0.01 k	0.14 ± 0.06 abcde	42 ± 3 bcd	0.37 ± 0.01 a
	6	13 ± 1 bcd	0.26 ± 0.02 ef	5.91 ± 0.15 cd	0.39 ± 0.01 h	0.45 ± 0.01 i	0.13 ± 0.01 abcd	ND	ND
	8	15 ± 1 bcde	0.28 ± 0.01 f	7.35 ± 0.19 de	0.44 ± 0.01 ij	0.50 ± 0.01 k	0.15 ± 0.01 abcdef	ND	ND
	10	21 ± 1 def	0.29 ± 0.01 f	10.22 ± 0.75 f	0.44 ± 0.01 ij	0.50 ± 0.01 jk	0.16 ± 0.01 abcdefg	ND	ND
*Analysis of variance and significance (p-values)*									
Type (A)		***	***	***	***	***	***	***	***
Concentration (B)		***	***	***	***	***	***	***	***
A × B		***	***	***	***	***	***	***	***

D01S, D24S, WHTS, and REDS, respectively, represent Desta 01, Desta 24, white, and red anchote cultivar starches. PS = potato starch and CS = cassava starch. Data are the mean ± standard deviation (*n* = 3). Values in the same column followed by the same letter are not significantly different (*p* < 0.05). G_1_′, G_1_″, and (tan δ)_1_, respectively, stand for the elastic modulus, viscous modulus, and loss tangent at a frequency of 1 Hz. a, b, and c are exponents showing the extent of dynamic moduli and the loss tangent on the frequency of oscillation. τ_max_ = the maximum stress that the samples can tolerate in the LVR. The cross point represents the stress at which G′ = G″. ND = not detectable. Analysis of variance and significance: *** = *p* < 0.001.

**Table 3:** Kinetics of texture evolution for anchote cultivars (D01S = Desta 01 starch, D24S = Desta 24 starch, WHTS = white starch and REDS = red starch) and potato starch (PS) gels stored at 4 °C (for 0, 8, 24, 48, 96, 144, and 192 h). Values of Avrami model factors.

Sample	*P*_0_ (N)	*P*_∞_ (N)	*k* (h^−n^)	*n*	*t*_1/2_ (h)
Firmness					
D01S	2.302 ± 0.259 ab	9.76 ± 0.149 b	0.054 ± 0.014 c	0.690 ± 0.045 a	41.89 ± 5.58 a
D24S	3.472 ± 0.644 c	10.267 ± 0.372 b	0.053 ± 0.010 c	0.654 ± 0.007 a	53.18 ± 12.78 ab
WHTS	2.679 ± 0.373 bc	13.484 ± 0.113 c	0.005 ± 0.003 a	1.169 ± 0.108 b	67.38 ± 6.31 b
REDS	3.119 ± 0.180 bc	13.350 ± 0.063 c	0.033 ± 0.012 bc	0.809 ± 0.109 a	45.19 ± 2.14 a
PS	1.628 ± 0.182 a	7.730 ± 0.416 a	0.009 ± 0.005 ab	1.115 ± 0.104 b	52.88 ± 7.93 ab
Springiness					
D01S	0.801 ± 0.024 a	0.563 ± 0.034 ab	3.0 × 10^−4^ ± 1.3 × 10^−4^ a	1.271 ± 0.003 b	72.49 ± 3.01 b
D24S	0.842 ± 0.027 a	0.480 ± 0.003 a	0.017 ± 0.003 b	1.187 ± 0.029 b	22.80 ± 1.04 a
WHTS	0.813 ± 0.004 a	0.687 ± 0.068 b	0.005 ± 0.002 a	1.614 ± 0.177 c	21.74 ± 1.17 a
REDS	0.850 ± 0.056 a	0.543 ± 0.051 ab	1.14 × 10^−4^ ± 0.16 × 10^−4^ a	1.919 ± 0.037 c	93.81 ± 1.31 c
PS	0.838 ± 0.042 a	0.641 ± 0.114 ab	1.40 × 10^−2^ ± 0.02 × 10^−2^ b	0.842 ± 0.003 a	100.97 ± 0.15 d
Cohesiveness					
D01S	0.637 ± 0.001 a	0.104 ± 0.001 a	0.006 ± 0.004 a	1.276 ± 0.205 a	47.38 ± 0.57 b
D24S	0.637 ± 0.015 a	0.133 ± 0.007 b	0.017 ± 0.024 a	1.951 ± 1.375 a	24.71 ± 4.28 a
WHTS	0.616 ± 0.031 a	0.136 ± 0.012 b	0.004 ± 2.0 × 10^−5^ a	1.320 ± 0.035 a	50.99 ± 5.52 b
REDS	0.652 ± 0.013 a	0.260 ± 0.002 c	0.009 ± 0.001 a	1.063 ± 0.016 a	61.92 ± 4.49 b
PS	0.634 ± 0.011 a	0.321 ± 0.004 d	3.30 × 10^−4^ ± 0.14 × 10^−4^ a	1.668 ± 0.011 a	97.81 ± 5.40 c
Resilience					
D01S	0.658 ± 0.007 a	0.081 ± 0.033 a	0.017 ± 0.002 a	1.082 ± 0.298 a	40.52 ± 2.61 b
D24S	0.638 ± 0.001 a	0.089 ± 0.001 a	2.00 × 10^−3^ ± 0.10 × 10^−3^ a	2.017 ± 0.087 b	18.83 ± 1.59 a
WHTS	0.628 ± 0.039 a	0.094 ± 0.020 a	1.97 × 10^−4^ ± 0.14 × 10^−4^ a	1.887 ± 0.017 b	75.79 ± 0.02 c
REDS	0.683 ± 0.015 a	0.247 ± 0.010 b	6.10 × 10^−5^ ± 0.83 × 10^−5^ a	2.032 ± 0.021 b	98.89 ± 1.85 d
PS	0.670 ± 0.031 a	0.298 ± 0.007 c	2.25 × 10^−5^ ± 0.25 × 10^−5^ a	2.291 ± 0.290 b	111.57 ± 5.80 e
Adhesiveness					
D01S	0.910 ± 0.042 cd	0.600 ± 0.010 ab	2.00 × 10^−3^ ± 0.03 × 10^−3^ c	1.401 ± 0.018 b	68.41 ± 4.38 d
D24S	0.770 ± 0.014 c	0.680 ± 0.014 c	1.80 × 10^−2^ ± 0.04 × 10^−2^ e	1.204 ± 0.013 a	20.59 ± 0.31 b
WHTS	0.395 ± 0.007 a	0.550 ± 0.057 a	3.40 × 10^−3^ ± 0.12 × 10^−3^ d	2.615 ± 0.003 d	7.64 ± 0.10 a
REDS	0.565 ± 0.007 b	0.660 ± 0.028 bc	1.30 × 10^−4^ ± 0.10 × 10^−4^ a	2.774 ± 0.007 e	22.01 ± 0.45 b
PS	0.970 ± 0.113 d	0.730 ± 0.014 c	1.00 × 10^−3^ ± 0.12 × 10^−3^ b	1.735 ± 0.009 c	41.23 ± 3.46 c
Gumminess					
D01S	1.566 ± 0.023 a	1.143 ± 0.026 a	9.19 × 10^−6^ ± 5.44 × 10^−6^ a	2.392 ± 0.135 c	113.85 ± 0.30 c
D24S	2.216 ± 0.462 a	1.576 ± 0.006 b	0.026 ± 0.002 b	1.042 ± 0.022 a	23.20 ± 0.39 a
WHTS	2.044 ± 0.419 a	1.525 ± 0.095 b	3.15 × 10^−6^ ± 0.39 × 10^−6^ a	2.682 ± 0.015 cd	98.26 ± 2.02 b
REDS	2.935 ± 0.017 b	1.678 ± 0.142 b	1.27 × 10^−4^ ± 0.33 × 10^−4^ a	1.838 ± 0.050 b	108.76 ± 1.81 c
PS	2.134 ± 0.006 a	1.477 ± 0.031 b	2.32 × 10^−7^ ± 1.22 × 10^−7^ a	2.992 ± 0.097 d	149.61 ± 3.40 d
Chewiness					
D01S	1.172 ± 0.096 a	0.634 ± 0.038 a	1.92 × 10^−5^ ± 0.03 × 10^−5^ a	2.254 ± 0.037 b	105.50 ± 7.35 b
D24S	1.872 ± 0.448 b	0.701 ± 0.081 a	0.018 ± 0.002 b	1.090 ± 0.031 a	28.12 ± 0.45 a
WHTS	1.536 ± 0.171 ab	1.175 ± 0.205 b	9.15 × 10^−6^ ± 0.96 × 10^−6^ a	2.509 ± 0.275 b	103.21 ± 3.35 b
REDS	2.834 ± 0.107 c	1.726 ± 0.055 c	3.02 × 10^−5^ ± 0.93 × 10^−5^ a	2.125 ± 0.055 b	113.93 ± 2.81 bc
PS	1.216 ± 0.006 a	0.923 ± 0.103 ab	2.16 × 10^−5^ ± 0.06 × 10^−5^ a	2.153 ± 0.010 b	123.89 ± 0.99 c

D01S, D24S, WHTS and REDS, respectively, represent Desta 01, Desta 24, white and red anchote cultivar starches. PS = potato starch. The data shown are the regression coefficients ± confidence interval. The values in the same column with different letters are significantly different (*p* < 0.05).

## Data Availability

Data will be availed by the authors upon request.
